# *MIR210HG* promotes breast cancer progression by IGF2BP1 mediated m6A modification

**DOI:** 10.1186/s13578-022-00772-z

**Published:** 2022-03-28

**Authors:** Wenjing Shi, Yongzhe Tang, Jing Lu, Yihui Zhuang, Jie Wang

**Affiliations:** 1 Department of Breast Diseases, The International Peace Maternity and Child Health Hospital, School of Medicine, Shanghai Jiao Tong University, Shanghai, People’s Republic of China; 2 Shanghai Key Laboratory of Embryo Original Diseases, Hengshan Rd. 910, Shanghai, 200030 China; 3 Experimental and Molecular Pathology, Institute of Pathology, Ludwig-Maximilians-University, Munich, Germany; 4 Shanghai Municipal Key Clinical Speciality, Shanghai, China; 5 Department of Clinical Laboratory, Fudan University Shanghai Cancer Center, Shanghai, China; 6 Department of Oncology, Shanghai Medical College, Fudan University, Shanghai, China

**Keywords:** *MIR210HG*, miR-210, IGF2BP1, MYCN, m6A, Breast cancer

## Abstract

**Background:**

Breast cancer is the most common cancer in women around the world, and the molecular mechanisms of breast cancer progression and metastasis are still unclear. This study aims to clarify the function and N6,2′-O-dimethyladenosine (m6A) regulation of lncRNA *MIR210HG* in breast cancer.

**Results:**

High expression of *MIR210HG* was confirmed in breast cancer. *MIR210HG* promoted breast cancer progression, which was mediated by its encoded miR-210. *MIR210HG* was regulated by IGF2BP1 mediated m6A modification. IGF2BP1 was confirmed highly expressed in breast cancer and induced both *MIR210HG* and miR-210 expression, which contributed to breast cancer progression. In addition, *MIR210HG* transcript was stabilized by IGF2BP1 and co-factor ELAVL1. *IGF2BP1* was a direct target of MYCN via E-box binding motif. MYCN induced IGF2BP1 expression in breast cancer cells. *MIR210HG* and miR-210 expressions were also increased by MYCN.

**Conclusions:**

In breast cancer, *MIR210HG* functions as an oncogenic lncRNA, which is also mediated by its encoded miR-210. In addition, both IGF2BP1 and ELAVL1 enhance the stability of *MIR210HG*, which contributes to the progression of breast cancer. Interestingly, *IGF2BP1* is directly activated by MYCN, which explains the oncogenic role of MYCN. These findings clarify the m6A regulation related molecular mechanism of breast cancer progression. The MYCN/IGF2BP1/*MIR210HG* axis may serve as an alternative molecular mechanism of breast cancer progression.

**Supplementary Information:**

The online version contains supplementary material available at 10.1186/s13578-022-00772-z.

## Introduction

Breast cancer is the most common cancer in women around the world [1]. Although therapies have been improved in past few decades, the 5-year overall survival rate of patients is largely decreased once metastasis and drug resistance happen [2]. However, the molecular mechanisms of breast cancer progression and metastasis are still unclear, which conceives obstacles in breast cancer treatment. In recent years, studies have shown that long non-coding RNA (lncRNA) plays critical roles in breast cancer progression [3, 4], which greatly supplements the gene regulation in this disease. LncRNAs execute their functions via various of paths such as transcription regulation, competitive endogenous RNA regulation and N6-Methyladenosine (m6A) modification [5]. In this study, the m6A regulation on lncRNA will be demonstrated.

The lncRNA *MIR210HG* is a host gene encoding miR-210, located in 21q13.3 with 567 nucleotides [6]. Previous studies reveal that *MIR210HG* functions as an oncogene and promotes tumor progression. In endometrial cancer, *MIR210HG* enriches genes of Wnt and TGF-β/Smad3 signaling pathways, which reinforces cancer development [7]. In breast cancer, *MIR210HG* is involved in Warburg Effect that induces tumor growth [8]. Furthermore, *MIR210HG* strengthens metastasis of breast cancer by forcing mucin-1 expression through ceRNA regulation [9]. Nevertheless, the more detailed and subtle molecular mechanisms and regulation model of *MIR210HG* in breast cancer is largely unknown. Thus, our study will illustrate the regulation of *MIR210HG* in breast cancer progression.

Besides being involved in transcription complex and ceRNA regulation, lncRNA is also mediated by m6A modification. In many cases, m6A locus contains “RRACH” motif and is associated with RNA abundance and stability [10]. In pathological processes, m6A methylation is regulated by methyltransferases (“writers”), demethylases (“erasers”) and binding proteins (“readers”) [11]. Studies validate that methyltransferases such as METTL3, METTL14 play dominant roles in cancer metastasis via m6A methylation [12, 13]. Demethylases such as FTO and ALKBH5 reversed the action of methyltransferases. FTO affects mRNA transcript levels via modulating RNA degradation in breast cancer [14]. As m6A readers, RNA-binding proteins including YTHDF1/2/3, IGF2BP1/2/3 and HNRNPC recognize and bind to m6A sites. Among these “readers”, IGF2BP1/2/3 are mainly related to RNA stability [15]. Interestingly, IGF2BPs preferentially recognize and bind to the m6A sites of oncogene *MYC* in the region of the coding region instability determinant (CRD) [16], which results in enhanced *MYC* expression, illustrating IGF2BPs promote tumorigenesis. However, the m6A regulation between IGF2BPs and *MIR210HG* in breast cancer is uncovered. Therefore, it is intriguing to reveal the effect of m6A regulation on *MIR210HG* in breast cancer and provide expanded molecular mechanisms in cancer progression.

In this study, we show that *MIR210HG* is an oncogenic lncRNA which promotes breast cancer progression. In addition, IGF2BP1 mediated m6A regulation induces *MIR210HG* expression. Interestingly, ELAVL1, a co-factor of IGF2BP1 is also involved in maintaining stability of *MIR210HG*. MYCN is confirmed to induce IGF2BP1/*MIR210HG* as a transcription activator of IGF2BP1. Therefore, the MYCN/IGF2BP1/*MIR210HG* axis may serve as an alternative molecular mechanism of breast cancer progression.

## Materials and method

### Cell culture

Breast cancer cell lines MCF-7, SK-BR-3, MDA-MB-231, MDA-MB-435 and MDA-MB-436 cells were cultivated in DMEM medium containing 10% % fetal bovine serum (FBS) (Invitrogen, Carlsbad, CA), 100 units/ml penicillin and 0.1 mg/ml streptomycin in 20% O2, 5% CO_2_ and 37℃. In transfection, miR-210 inhibitor and siRNA pools were transfected by LipoRNAiMAX Transfection Reagent (Invitrogen) according to product instructions. Inducible vector pLVX-MIR210HG-Tet-On3G and pLVX-MYCN-HA-Tet-On3G (designed and constructed by Maokang Bio, Shanghai, China) were transfected with Lipofectamine LTX Reagent (Invitrogen) based on product instructions. The final concentrations of RNAs and vectors were 25 nM and 2 μg/ml respectively. Puromycin with 2 μg/ml concentration was maintained to keep cells continuously expressing inducible vector. To induce *MIR210HG* or MYCN-HA expression, 50 ng/ml Dox was added into medium. MCF-7 and MDA-MB-231 cells with inducible vectors were named as MCF-7^*MIR10HG*−OV^, MCF-7^*MYCN*−HA−OV^, 231^*MIR210HG*−OV^ and 231^*MYCN*−HA−OV^. To achieve IGF2BP1 overexpression, a pcDNA3.1-IGF2BP1 vector was designed and constructed by Maokang Bio (Shanghai, China). The final transfection concentration of this vector was 2 μg/ml in a 6-well plate.

### Human samples sources

Tissues from breast cancer patients (n = 10) within last 5 years and paraffin-embedded tissue sections (n = 6) collected between year 2015–2018 were obtained from The International Peace Maternity & Child Health Hospital of China welfare institute (IPMCH), affiliated to Shanghai Jiaotong University. All separated tissues were stored in liquid nitrogen before performing experiments. This this study was approved by the Ethics Committee of IPMCH. All patients signed informed consent forms.

### RNA isolation and quantitative real-time PCR (q-PCR)

Total RNAs in tissues and cells were isolated by RNeasy Micro Kit (QIAGEN) and High Pure RNA Isolation Kit (Roche) respectively according to the manufacturers’ instructions. For reverse transcription, tissue RNA was used by employing PrimeScript RT reagent Kit (Takara, Dalian, China) with gDNA Eraser to remove genomic DNAs. Cell RNA was reverse transcribed with Verso cDNA Synthesis Kit (Thermo Fisher, USA). Q-PCR was performed with SYBR Green Master Mix (Thermo Fisher) in 7500 Real-Time PCR System (Thermo Fisher). Gene expression was normalized to *GAPDH* and calculated with 2^(-∆∆Ct) method. Primers used were listed in Additional file 1: Table S1. Specially, for mRNA stability measurement, cells were treated with Actnomycin D (5 μM) to arrest transcription or DMSO as a negative control after 48 h of indicated transfections. RNA was collected at each designated time points and performed by q-PCR analysis. For quantification, the contents of RNA (Actnomycin D)/ RNA (DMSO) were converted to percentage of RNA at 0 h (100%).

### Western blot analysis

Total protein of cells was extracted by RIPA lysis buffer. Protein concentration was determined by BCA Protein Assay Kit (TIANGEN, China). In each group, 30 μg protein was loaded and separated by SDS-PAGE gels and then transferred to PVDF membrane (Millipore, Massachusetts, USA). Membranes were block with 5% BSA dissolved in TBST for 30 min at room temperature. Then membranes were incubated with primary antibodies at 4 ℃ overnight. Secondary antibody was incubated with membrane for 1 h at room temperature. Antibodies used were listed in Additional file 1: Table S2.

### Colony formation assay

Cells were seeded into 6-well plate at the density of 300 cells/well and cultivated for 3–4 weeks until obvious colonies can be seen by eyes. Medium was replaced every 5 days. Then cells were fixed with 4% PFA (v/v in PBS) for 30 min at room temperature. 0.5% crystal violet was used to stain cells for 20 min. Wash cells under the running water and let it dry in air flow. Pictures were taken by Digital camera (NIKON) and cells were counted by Image J software via measuring colony number.

### Migration and invasion assay

For migration ability measurement, scratch assay was employed. Cells were seeded into 6-well plate and cultivated overnight. Cell surface wound was generated by scratching with a 10 μl tip. Cell debris were removed by pre-warmed PBS and then cultivated for 1 h. Wound images were taken after 1 h cultivation and recorded as 0 h timepoint. Cells were continuously cultivated for 24 h and then take images recorded as a terminal timepoint. Migration rate was calculated with the difference of distances between two timepoints and normalized to control group.

For invasion assay, cells were seeded into the upper chamber coated with Matrigel (Corning, USA) at the density of 1 × 10^5^ cells/well. FBS free DMEM medium was filled in upper chamber while DMEM medium containing 10% FBS was used in the bottom chambers. After 48 h, chambers were taken out and cells were fixed by methanol for 30 min. 0.5% crystal violet was used to stain cells for 20 min. Gently removed cells in upper chamber and washed whole chamber 3 times with PBS. Images were taken by microscope. Quantification was performed with Image J software to count cell number.

### M6A-RNA immunoprecipitation (MeRIP) assay

Total RNA was extracted from MCF-7 and MDA-MB-231 cells, and treated with DNase (Sigma) to remove genomic DNA. After mRNA purification and fragmentation, the fragments were incubated with m6A primary antibody for immunoprecipitation using a Magna MeRIP™ m6A kit (#17–10,499, Merck Millipore, MA, USA). Enriched m6A modified mRNA was then detected by q-PCR.

### Luciferase assay

The sequences of *IGF2BP1* promoter regions with wild type or mutant E-box were inserted to pBV-Luc vector (Addgene, USA), named as IGF2BP1 WT and IGF2BP1 MUT respectively. The experiments were conducted following manufacturer’s instruction (Promega, USA). Primers used were listed in Additional file 1: Table S3.

### ChIP-qPCR (q-ChIP)

MCF-7 and MDA-MB-231 cells were used to confirm the transcription factor target. Crosslinking process and chromatin immunoprecipitation were performed according to the instruction provided in the QuikChIP(TM) Kit (NOVUS, USA). Q-PCR was conducted followed ChIP. Antibodies were listed in Additional file 1: Table S2 and primers were listed in Additional file 1: Table S4.

### Immunohistochemistry (IHC)

The specimens were paraffin-embedded. Before experiments, the tissue sections (3 μm) were dewaxed, rehydrated, and blocked with 3% BSA and subjected to antigen retrieval. After washing, the sections were incubated primary antibodies at 4 °C overnight. Mouse IgG was then used as the negative control. After incubation, the bound antibodies were detected using horseradish peroxidase (HRP)-conjugated goat anti-rabbit IgG or goat anti-mouse IgG and visualized by diaminobenzidine (DAB). The results were graded according to both the intensity and the percentage of positive cells under a microscope by two pathologists in a blinded manner. Antibodies used were listed in Additional file 1: Table S2.

### Co-immunoprecipitation (Co-IP) analysis

For Co-IP analysis, cell lysates were resuspended in RIPA buffer containing protease and phosphatase inhibitors (Roche, Switzerland). Protein supernatant was collected by centrifuging at 12,000 rpm for 30 min at 4 ℃. The IP reaction was performed overnight at 4℃ with IGF2BP1 antibody or control IgG. The precipitates were incubated with Protein A-Sepharose beads at 4 ℃ for 2–4 h. The precipitates were washed 3 times with RIPA buffer and finally resuspended in 4 × Laemmli buffer for western blot analysis. Antibodies used in Co-IP were listed in Additional file 1: Table S2.

### Animal experiments

Female BALB/c nude mice (4-week-old) were purchased from Shanghai Model Organisms Center, Inc. and housed under standard conditions. In the tumor growth xenograft model, 5 × 10^6^ MDA-MB-231 cells with scrambled siRNA and indicated siRNAs transfected were suspended in 100 μl serum-free DMEM and injected into the right flank of each mouse subcutaneously. 231^*MIR210HG*−OV^ and 231^*MYCN*−HA−OV^ cells with indicated treatments were also suspended in 100 μl serum-free DMEM and injected into the right flank of each mouse subcutaneously. The volumes of tumors were measured and calculated as 0.5 × length × width^2^. After 30 days the mice were killed and the tumors were removed for further analysis such tumor weight measurement.

### Bioinformatics analysis

The data set used is from GEO database (https://www.ncbi.nlm.nih.gov/geo/), and the download data format is MINIML. Limma package (version: 3.40.2) of R software was used to study the differential expression of mRNAs. The box plot is implemented by the R software package ggplot2. Expression data and clinical data of related mRNAs was analyzed from TCGA-BRCA datasets downloaded from the National Cancer Institute’s Genomic Data Commons (https://gdc.cancer.gov/). M6A modification prediction was performed through http://m6a2target.canceromics.org/#/home and m6A-Atlas (http://180.208.58.66/m6A-Atlas/index.html). The data of ChIP-Seq analysis was obtained from Cistrome DB (http://cistrome.org/db/#/) and the scheme was generated in UCSC genome browser.

### Statistics

Student’s t test (2-tailed; unpaired) was performed and calculated the significant differences between two groups. In multiple groups comparisons, 1-way analysis of variance followed by a Tukey multiple comparisons post hoc test was used. P values less than 0.05 was considered as a significant difference (*p < 0.05, **p < 0.01, ***p < 0.001, ****p < 0.0001). Statistics were performed with Prism 8 (GraphPad Software, USA).

## Results

### *MIR210HG* is highly expressed in breast cancer

**Fig. 1:**
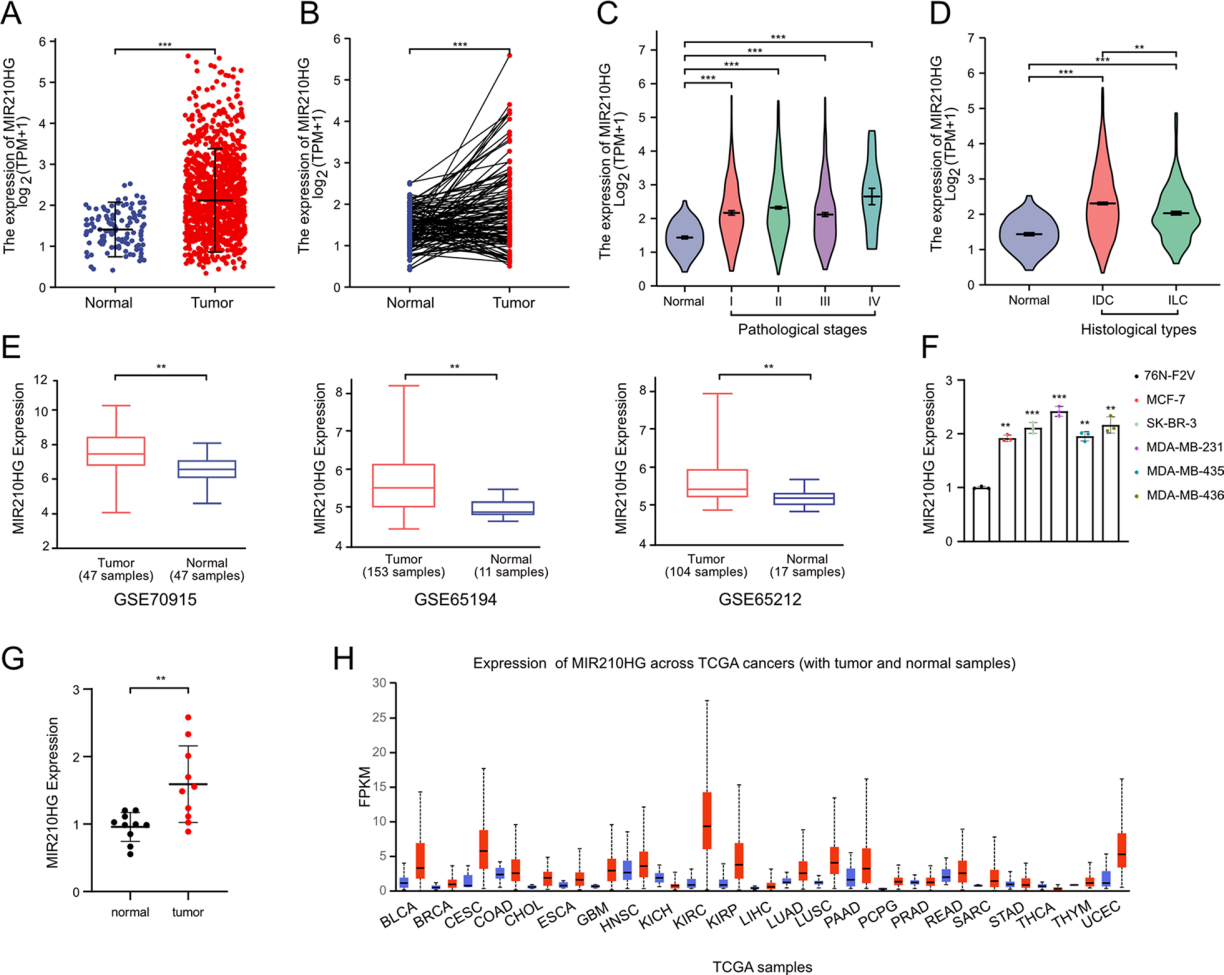
*MIR210HG* is highly expressed in breast cancer. **A** and **B** Unpaired and paired analysis of *MIR210HG* expression in TCGA-BRCA. In unpaired analysis, 1109 tumor samples and 113 normal samples were included. In paired analysis, 112 tumor samples and 112 normal samples were included. **C** The expression of *MIR210HG* in different pathological stages was analyzed based on TCGA-BRCA dataset. **D** The expression of *MIR210HG* in Invasive Ductal Carcinoma (IDC) and Invasive lobular Carcinoma (ILC) was analyzed and compared to normal tissue. **E** *MIR210HG* expression was analyzed in three independent GEO datasets including GSE70915, GSE65194 and GSE65212. **F** q-PCR analysis of *MIR210HG* expression in five indicated breast cancer cell lines and compared to normal breast epithelium cell 76 N-F2V cell. **G** q-PCR analysis of *MIR210HG* expression in tumor tissues obtained from 10 patients and compared to adjacent normal tissues. **H** Pan-cancer analysis of *MIR210HG* expression. Data was obtained from TCGA datasets. Tumor samples were indicated as red bars and normal samples were indicated as blue bars. **p < 0.01, ***p < 0.001

By analyzing lncRNA *MIR210HG* expression profile in TCGA-BRCA datasets, we confirmed that *MIR210HG* was highly expressed in breast cancer tissues compared to normal tissues (Fig. 1A and B). In addition, high *MIR210HG* expression also indicated advanced pathological stages (Fig. 1C). In Invasive Ductal Carcinoma (IDC) and Invasive lobular Carcinoma (ILC), *MIR210HG* showed higher expression level compared to normal tissues (Fig. 1D). Then we performed analysis in three independent GEO datasets (GSE70905, GSE65194 and GSE65212) to further confirm *MIR210HG* expression profiles. High expression of *MIR210HG* was observed in breast cancer in all selected datasets (Fig. 1E). Next, q-PCR analysis was performed in 5 breast cancer cell lines including MCF-7, SK-BR-3, MDA-MB-231, MDA-MB-435 and MDA-MB-436. The results revealed that *MIR210HG* commonly had a significant high level in all 5 breast cancer cell lines compared to the normal breast epithelium cell 76 N-F2V (Fig. 1F). Moreover, we also tested *MIR210HG* expression in tissues from 10 breast cancer patients. In line with the results of cell lines, *MIR210HG* was observed to have a significant high expression level in breast cancer tissues compared to the adjacent parts (Fig. 1G). Of note, pan-cancer analysis indicated *MIR210HG* was highly expressed in most cancer types (Fig. 1H), suggesting that *MIR210HG* is an oncogene and promising target for cancer therapy. Collectively, *MIR210HG* is an oncogenic lncRNA, which is possibly involved in breast cancer progression.

### *MIR210HG* promotes proliferation of breast cancer

**Fig. 2:**
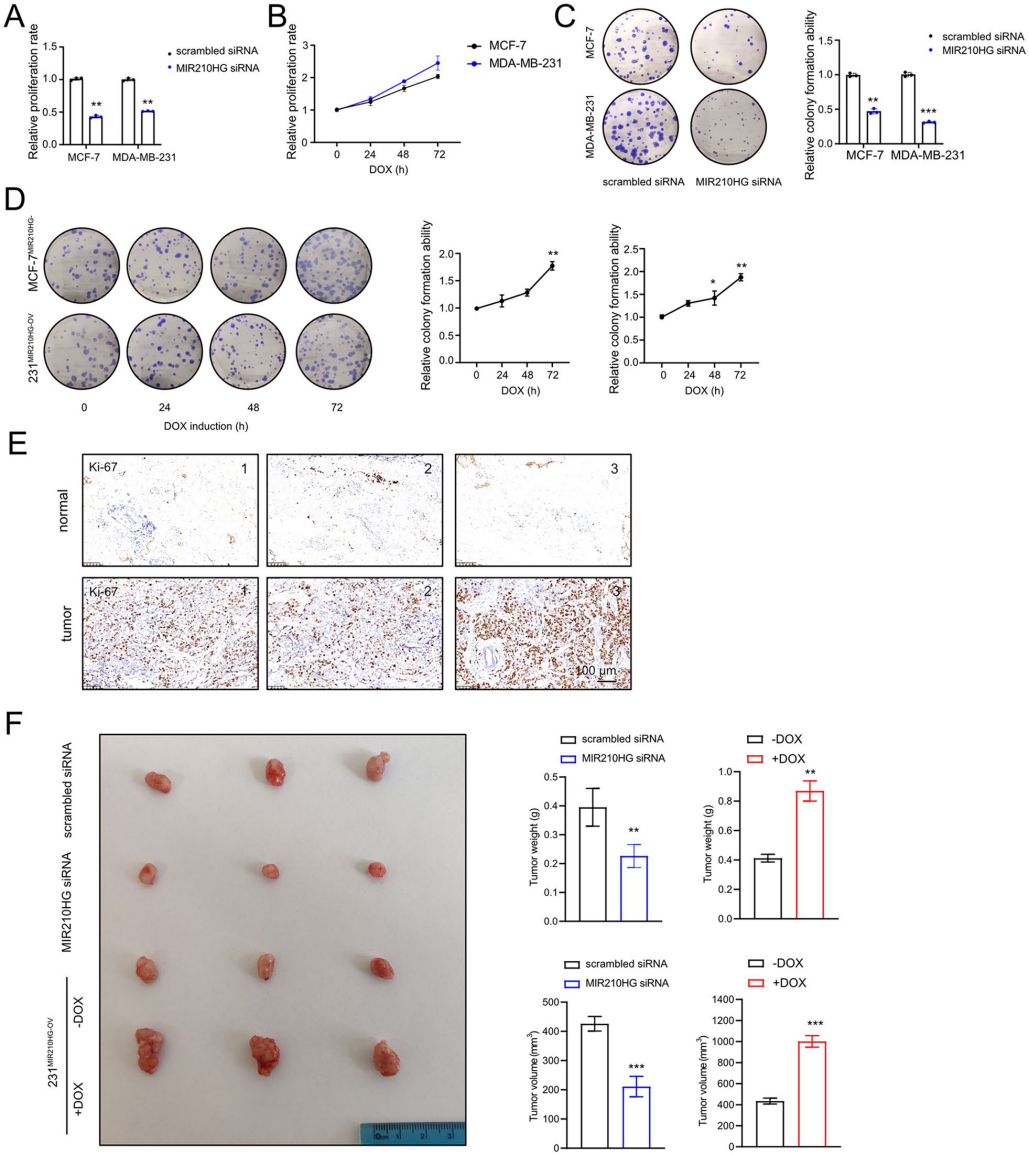
*MIR210HG* promotes proliferation of breast cancer. **A** Proliferation ability of MCF-7 and MDA-MB-231 cells were measured by MTT assay after transfected *MIR210HG* siRNA. The results were normalized to scrambled siRNA group. **B** Proliferation ability of MCF-7 and MDA-MB-231 cells were measured by MTT assay after *MIR210HG* was induced by DOX. The results were normalized to 0 h time point. **C** and **D** Colony formation assay analysis of MCF-7 and MDA-MB-231 cells after silencing and overexpression *MIR210HG* respectively. **E** Expression of Ki-67 was determined by immunohistochemistry (IHC) of 6 specimens of breast cancer patients. Scar bars: 100 μm. **F** Xenograft tumors of 4 groups: scrambled siRNA MDA-MB-231 cell; *MIR210HG* siRNA MDA-MB-231 cell; 231^*MIR210HG*−OV^ cell without DOX treatment; 231^*MIR210HG*−OV^ cell with DOX treatment. **p < 0.01, ***p < 0.001

Since *MIR210HG* was validated highly expressed in breast cancer, we continuously clarified *MIR210HG* functions. Firstly, *MIR210HG* was knocked down by siRNA or overexpressed by inducible vector in MCF-7 and MDA-MB-231 cells. MIR210HG overexpression cells were named MCF-7^*MIR210HG*−OV^ and 231^*MIR210HG*−OV^ respectively. Subsequently, MTT assay was employed to evaluate proliferation rates of breast cancer cells. Silencing *MIR210HG* repressed proliferation of breast cancer cells (Fig. 2A), whereas DOX induced *MIR210HG* increased breast cancer cell proliferation (Fig. 2B). Colony formation assay revealed that the colony forming capacity of breast cancer cells was prohibited by *MIR210HG* siRNA (Fig. 2C). Conversely, ectopic *MIR210HG* enhanced the colony forming capacity of breast cancer cells (Fig. 2D). In addition, we stained human breast cancer tissues with Ki-67, which indicated a high Ki-67 level in breast cancer tissues compared to adjacent tissues (Fig. 2E). Furthermore, in line with the in vitro results, in vivo analysis showed tumor growth was repressed by *MIR210HG* siRNA but induced by *MIR210HG* overexpression (Fig. 2F), evidenced by tumor weight and volume changes. Taken together, *MIR210HG* promotes breast cancer proliferation.

### *MIR210HG* promotes metastasis of breast cancer

**Fig. 3:**
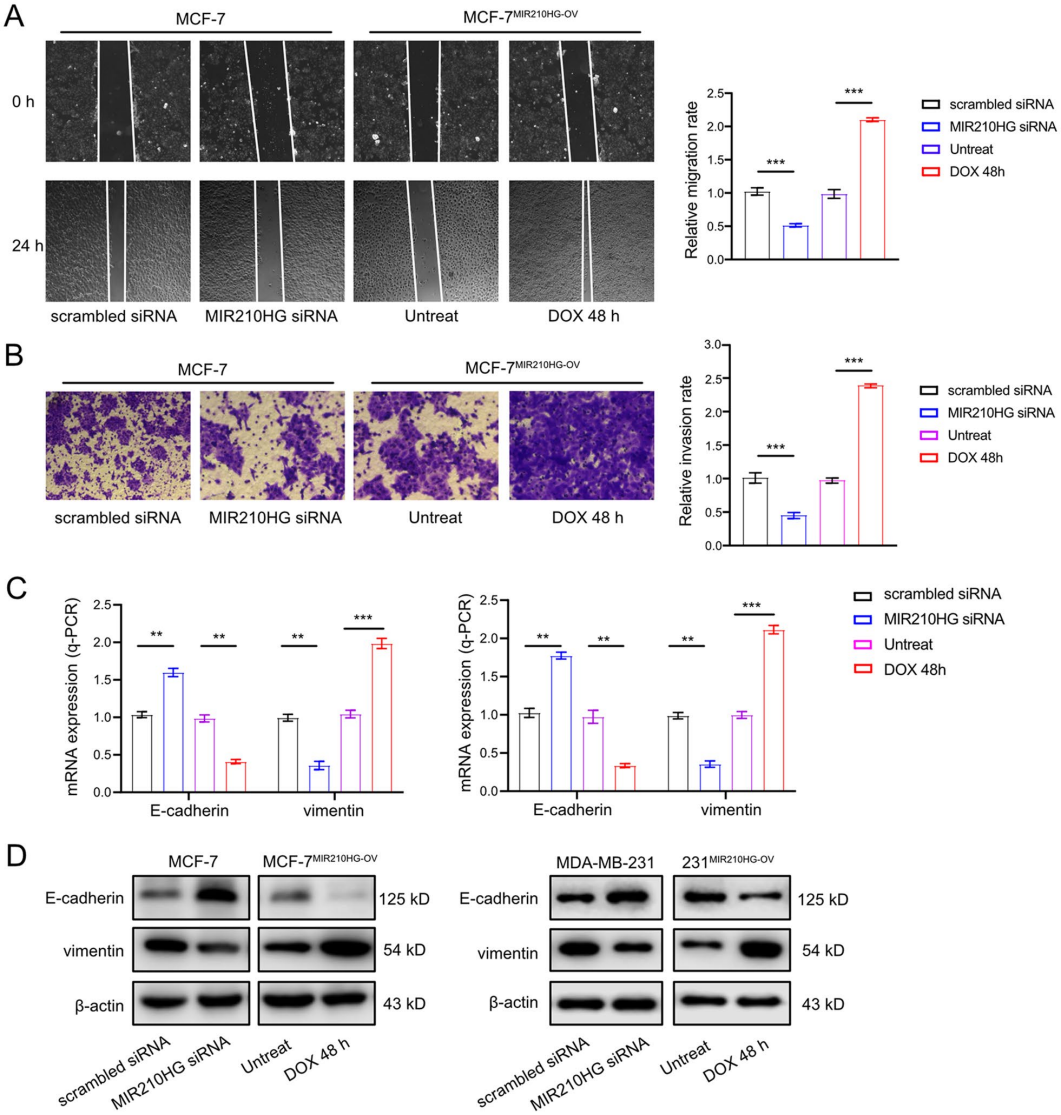
*MIR210HG* promotes metastasis of breast cancer. **A** Scratch assay of MCF-7 cell after MIR210HG silencing and MCF-7^*MIR210HG*−OV^ cell with DOX treatment. Quantification of scratch assay was also presented. The results were normalized to scrambled siRNA group and untreated group respectively. **B** Transwell assay of MCF-7 cell after *MIR210HG* silencing and MCF-7^*MIR210HG*−OV^ cell with DOX treatment. Quantification of transwell assay was also presented. The results were normalized to scrambled siRNA group and untreated group respectively. **C** and **D** EMT process markers E-cadherin and vimentin were tested by q-PCR and western blot in MCF-7 cell after *MIR210HG* silencing and MCF-7^*MIR210HG*−OV^ cell with DOX treatment. **p < 0.01, ***p < 0.001

In following step, we determined the function of *MIR210HG* in migration and invasion of breast cancer cells. Wound-healing assay results indicated that *MIR210HG* knocking down significantly repressed cell migration, whereas ectopic *MIR210HG* induced migration ability of MCF-7^*MIR210HG*−OV^ cell (Fig. 3A). In Transwell assay, silencing *MIR210HG* resulted in a lower invasion ability evidenced by less stained MCF-7 cells at the bottom of the chamber. Conversely, DOX induced *MIR210HG* promoted MCF-7^*MIR210HG*−OV^ cells to penetrate matrigel, indicating an enhanced invasion ability (Fig. 3B). Since Epithelial Mesenchymal Transition (EMT) is the main cause of migration and invasion, we investigated the EMT markers regulated by *MIR210HG* potentially. Q-PCR analysis showed that epithelial marker *E-cadherin* was increased while mesenchymal marker *vimentin* was repressed when *MIR210HG* was knocked down. *MIR210HG* overexpression resulted in a prohibited *E-cadherin* level but induced *vimentin* expression (Fig. 3C). Western blot results also showed similar results (Fig. 3D). Therefore, *MIR210HG* induces migration and invasion of breast cancer cells by regulating EMT process.

### miR-210 mediates *MIR210HG* function

**Fig. 4:**
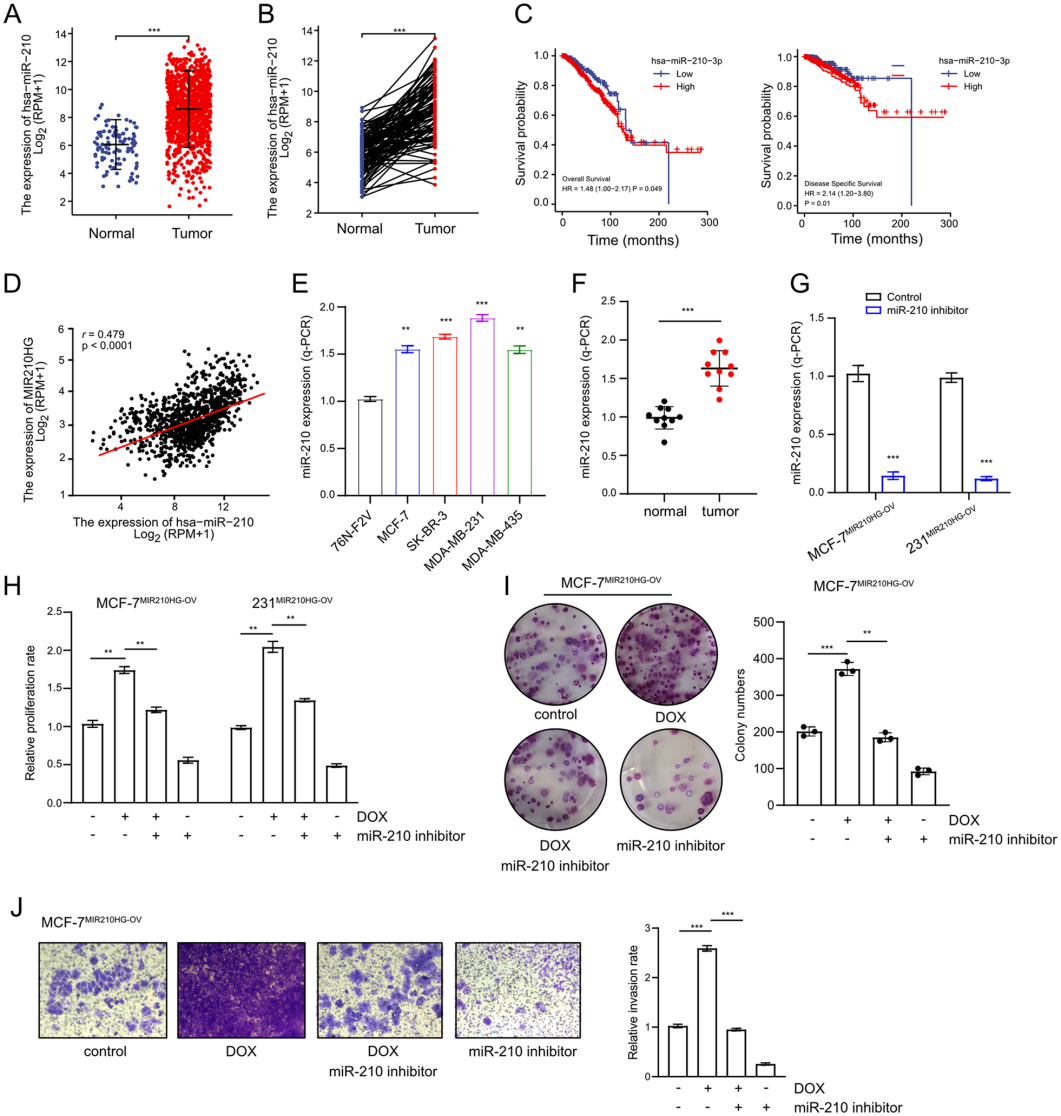
miR-210 mediates *MIR210HG* function. **A** and **B** Unpaired and paired analysis of miR-210 expression in TCGA-BRCA. In unpaired analysis, 1109 tumor samples and 113 normal samples were included. In paired analysis, 112 tumor samples and 112 normal samples were included. **C** Overall survival rate and distant metastasis free survival rate of breast cancer patients with different miR-210 expression level were analyzed based on TCGA-BRCA dataset. High miR-210 expression was indicated as a red line and low miR-210 expression was indicated as a blue line. **D** Pearson correlation analysis between *MIR210HG* expression and miR-210 expression in breast cancer patients. **E** q-PCR analysis of miR-210 expression in four indicated breast cancer cell lines compared to 76 N-F2V cell. **F** q-PCR analysis of miR-210 expression in breast cancer tissues compared to adjacent tissues from 10 patients. **G** q-PCR analysis on miR-210 expression in MCF-7^*MIR210HG*−OV^ and 231-7^*MIR210HG*−OV^ cells with miR-210 inhibitor treatment. **H** proliferation rate was analyzed by MTT assay in MCF-7^*MIR210HG*−OV^ and 231-7^*MIR210HG*−OV^ cells with indicated treatments. **I** Colony formation assay of MCF-7^*MIR210HG*−OV^ cells with indicated treatments. Quantification was presented. **J** Transwell assay of MCF-7^*MIR210HG*−OV^ cells with indicated treatments. Quantification was presented. **p < 0.01, ***p < 0.001

As *MIR210HG* is the host gene of miR-210, it is interesting to clarify whether miR-210 is required for *MIR210HG* function. By analyzing TCGA-BRCA datasets, miR-210 was confirmed to be highly expressed in breast cancer (Fig. 4A and B). In addition, high miR-210 expression indicated a poor overall survival rate and a poor distant metastasis free survival rate in breast cancer (Fig. 4C). Indeed, the expression of miR-210 was positively correlated to *MIR210HG* expression in breast cancer (Fig. 4D), suggesting an interaction in chain between miR-210 and *MIR210HG*. Furthermore, in five breast cancer cell lines, miR-210 was validated to have high expression levels compared to the normal breast epithelium cell 76 N-F2V (Fig. 4E). Similarly, high miR-210 was also confirmed in breast cancer patients (Fig. 4F). Next, to demonstrate miR-210 function, miR-210 was knocked down achieved by a specific miR-210 inhibitor in both MCF-7^*MIR210HG*−OV^ and 231^*MIR210HG*−OV^ cells (Fig. 4G). Interestingly, miR-210 inhibition largely abrogated proliferation rate which was enhanced by *MIR210HG* overexpression (Fig. 4H). In addition, colony formation capacity elevated by *MIR210HG* was also repressed by miR-210 inhibition (Fig. 4I). Similarly, miR-210 inhibitor prohibited invasion ability of MCF-7^*MIR210HG*−OV^, which was induced by *MIR210HG* (Fig. 4J). Collectively, miR-210 is an effector of *MIR210HG* and mediates *MIR210HG* function in breast cancer progression.

### *MIR210HG* is induced by IGF2BP1 mediated m6A modification

**Fig. 5:**
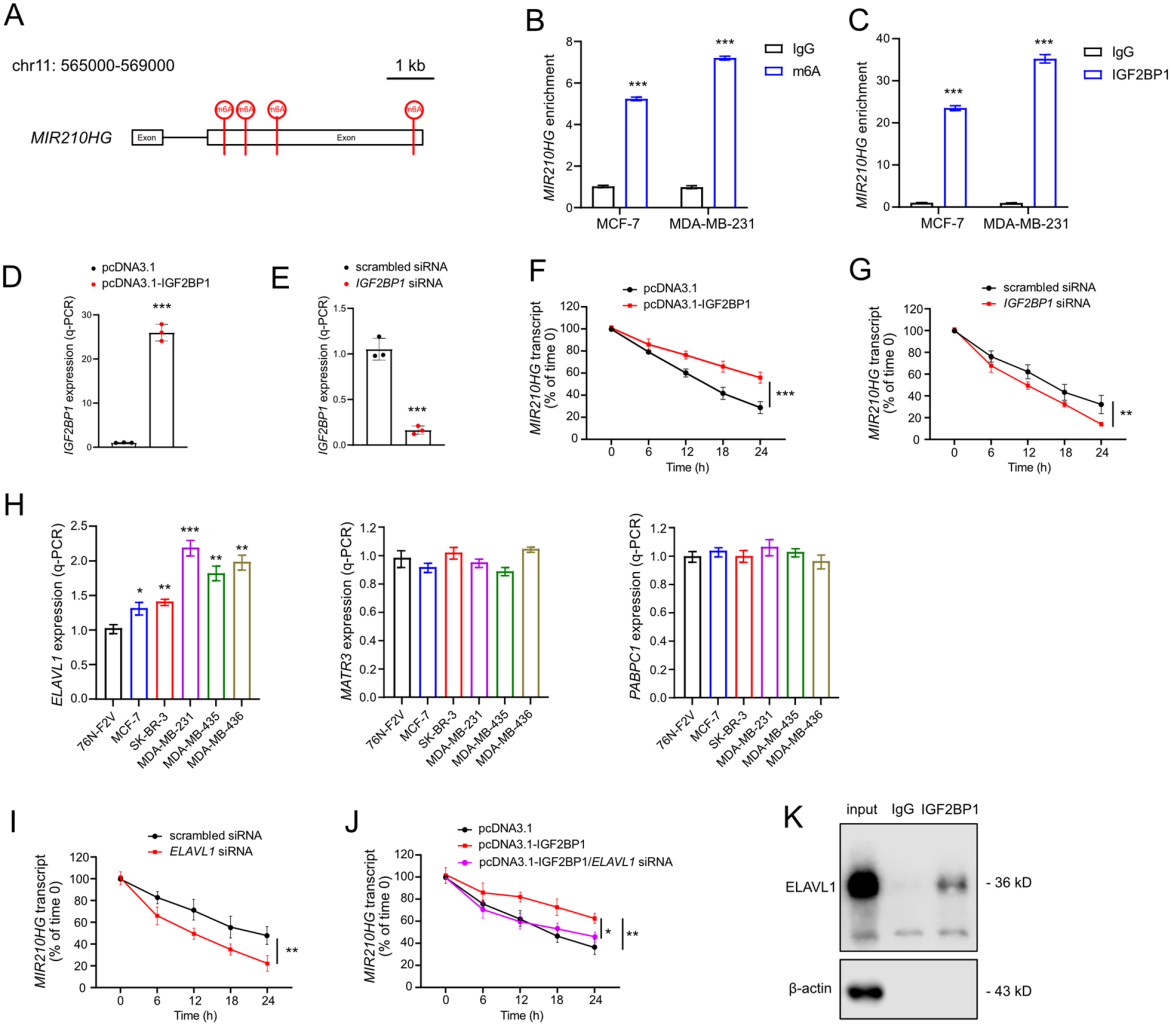
*MIR210HG* is induced by IGF2BP1 mediated m6A modification. **A** *MIR210HG* genome scheme of m6A modification sites. Four m6A sites were indicated. **B** and **C** MeRIP assays were performed to identify variation in m6A modification and IGF2BP1 enrichments in *MIR210HG* after overexpressing *MIR210HG* in breast cancer cells. **D** q-PCR analysis of ectopic expression of IGF2BP1 achieved by pcDNA3.1-IGF2BP1 vector transfection in MCF-7 cells. **E** q-PCR analysis of IGF2BP1 silencing achieved by *IGF2BP1* siRNA pools in MCF-7 cells. **F** and **G** the degradation of *MIR210HG* transcript was measured by q-PCR at different time points with indicated treatments. **H** q-PCR analysis of *ELAVL1*, *MATR3* and *PABPC1* in the indicated cell lines. **I** The degradation of *MIR210HG* transcript was measured by q-PCR at different time points after silencing *ELAVL1*. **J** The degradation of *MIR210HG* transcript was measured by q-PCR at different time points with indicated treatment. **K** Co-IP-western blot analysis of ELAVL1 by precipitation with IGF2BP1 antibody in MDA-MB-231 cell. *p < 0.05, **p < 0.01, ***p < 0.001

Accumulating evidences imply that m6A modification is one of the important epigenetic regulations on lncRNA functions. Therefore, it is interesting to investigate whether *MIR210HG* can be regulated through m6A modification. Through bioinformatics analysis, there were four potential m6A modification sites were searched (chr11:565778, chr11:567257, chr11:567410, chr11:567484), and m6A reader IGF2BP1 was predicted to bind and recognize the sites (Fig. 5A). Indeed, *MIR210HG* was enriched within the m6A sites in both MCF-7 and MDA-MB-231 cells (Fig. 5B). RIP results indicated that IGF2BP1 directly bind *MIR210HG* (Fig. 5C). Above results suggested that IGF2BP1 interacted with *MIR210HG* through m6A modification. Since IGF2BP1 functions an RNA binding protein, next we evaluated the stability of *MIR210HG* with ectopic *IGF2BP1* and silenced *IGF2BP1* (Fig. 5D and E). By overexpressing *IGF2BP1* and blocking new RNA synthesis, the degradation of *MIR210HG* was largely repressed (Fig. 5F). Conversely, silencing *IGF2BP1* induced the degradation of *MIR210HG* (Fig. 5G). Therefore, *MIR210HG* is regulated by IGF2BP1 through both m6A modification and transcript stabilization. Since previous studies identified that three known mRNA stabilizers including ELAV-like RNA binding protein 1 (ELAVL1; also known as HuR), matrin 3 (MATR3), and poly(A)-binding protein cytoplasmic 1 (PABPC1), are co-factors of IGF2BPs in enhancing stability of mRNA [16], here we also examined whether the three factors are involved in *MIR210HG* stabilization. In breast cancer cell lines, the expression of *ELAVL1* was up-regulated (Fig. 5H), indicating that ELAVL1 may serve as a co-factor of IGF2BP1 to stabilize *MIR210HG*. Interestingly, silencing *ELAVL1* induced the degradation of *MIR210HG* (Fig. 5I). In addition, silencing *ELAVL1* largely abrogated the effect of ectopic *IGF2BP1* on maintaining *MIR210HG* transcript (Fig. 5J). Furthermore, ELAVL1 was enriched in IGF2BP1 specific immunoprecipitation (Fig. 5K). Therefore, ELAVL1 is a co-factor of IGF2BP1, which contributes to the stabilization of *MIR210HG*.

**Fig. 6:**
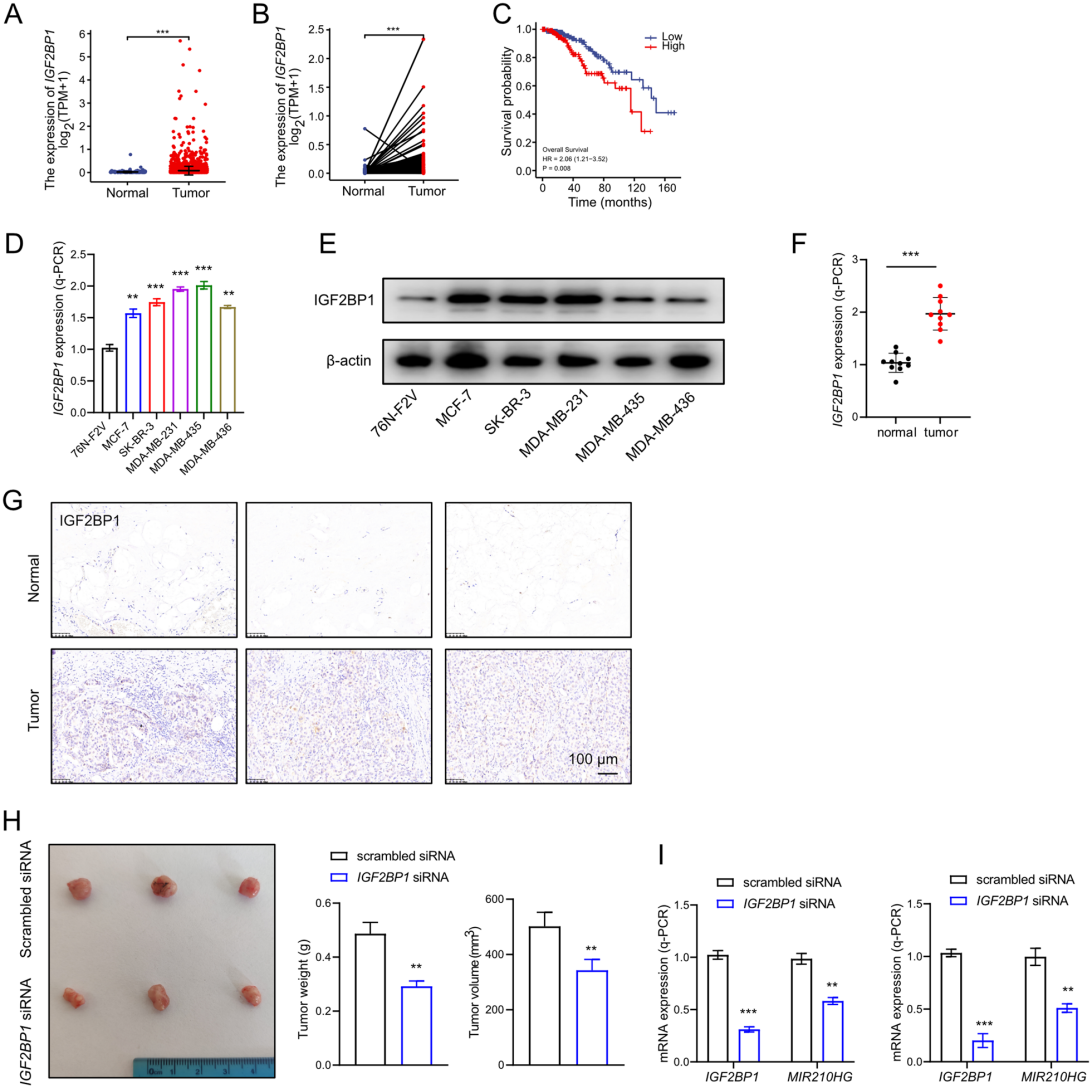
IGF2BP1 is up-regulated in breast cancer. **A** and **B** Unpaired and paired analysis of *IGF2BP1* expression in TCGA-BRCA. In unpaired analysis, 1109 tumor samples and 113 normal samples were included. In paired analysis, 112 tumor samples and 112 normal samples were included. **C** Overall survival rate of breast cancer patients with different *IGF2BP1* expression level were analyzed based on TCGA-BRCA dataset. High *IGF2BP1* expression was indicated as a red line and low *IGF2BP1* expression was indicated as a blue line. **D** and **E** q-PCR analysis and western blot analysis of miR-210 expression in five indicated breast cancer cell lines compared to 76 N-F2V cell. **F** q-PCR analysis of miR-210 expression in breast cancer tissues compared to adjacent tissues from 10 patients. **G** Expression of IGF2BP1 was determined by immunohistochemistry (IHC) of 6 specimens of breast cancer patients. Scar bars: 100 μm. **H** Xenograft tumors of 2 groups: scrambled siRNA MDA-MB-231 cell; *IGF2BP1* siRNA MDA-MB-231 cell. **I** q-PCR analysis on *MIR210HG* when *IGF2BP1* was silenced performed in breast cancer cells. **p < 0.01, ***p < 0.001

From analysis of TCGA-BRCA datasets, *IGF2BP1* was confirmed to be highly expressed in breast cancer (Fig. 6A and B). Furthermore, high *IGF2BP1* expression indicated a poor relapse survival rate (Fig. 6C). Q-PCR and western blot analysis indicated that IGF2BP1 was highly expressed in breast cancer cells compared to normal cell (Fig. 6D and E), implying that IGF2BP1 was positively correlated to breast cancer. In breast cancer patients, IGF2BP1 was also validated had a higher expression compared to adjacent normal tissues (Fig. 6F and G). Smaller tumors were observed in xenograft model injected with MDA-MB-231 cells transfected *IGF2BP1* siRNA (Fig. 6H). Silencing *IGF2BP1* significantly repressed *MIR210HG* expression in MCF-7 and MDA-MB-231 cells (Fig. 6I). In conclusion, IGF2BP1 induces *MIR210HG* through m6A modification, through which IGF2BP1 functions as an oncogene in breast cancer.

### MYCN regulates *MIR210HG* via IGF2BP1

**Fig. 7:**
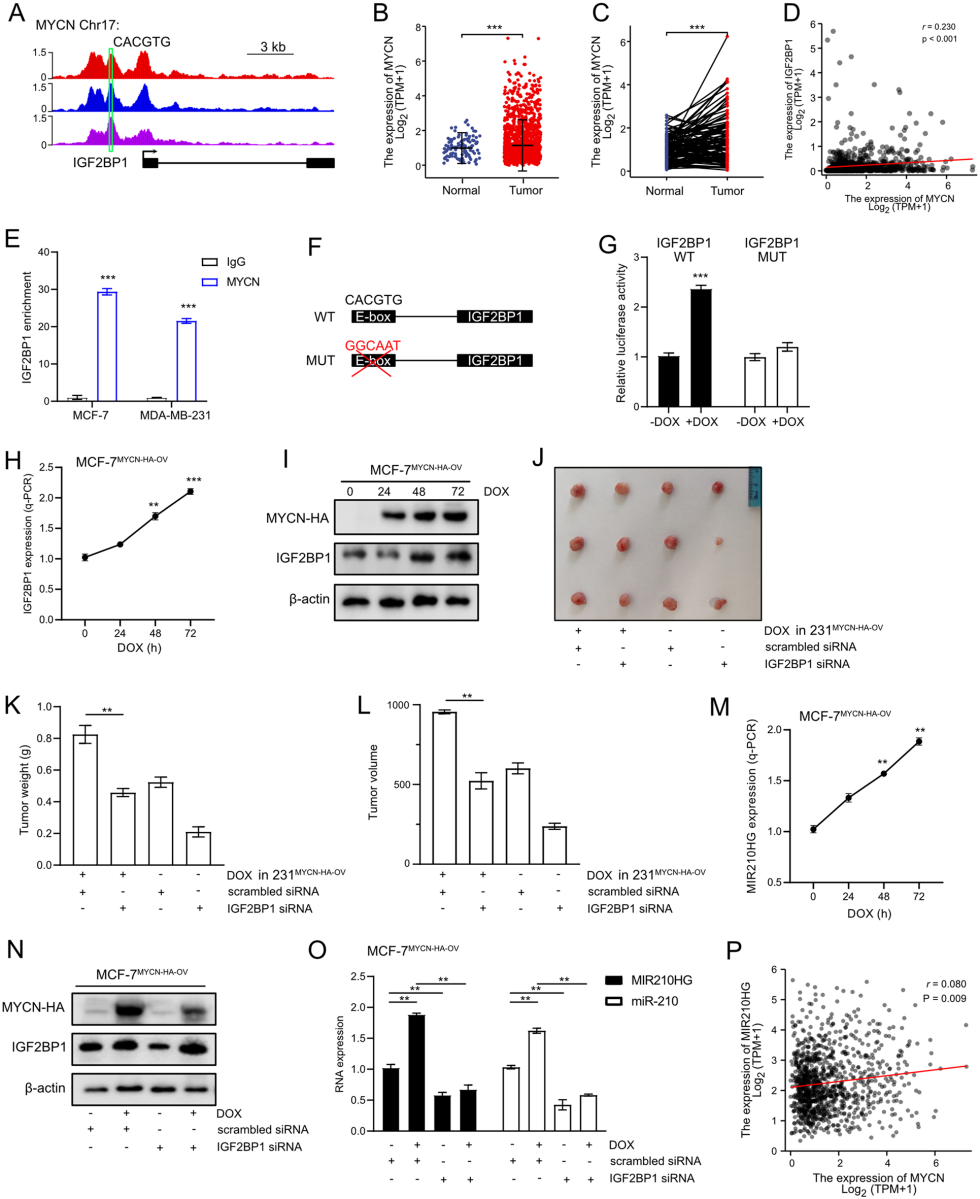
MYCN regulates *MIR210HG* via IGF2BP1. **A** MYCN ChIP-Seq analysis on *IGF2BP1* promoter. Three independent ChIP-Seq datasets were obtained from Cistrome DB. E-box was indicated in *IGF2BP1* genome scheme. **B** and **C** Unpaired and paired analysis of *MYCN* expression in TCGA-BRCA. In unpaired analysis, 1109 tumor samples and 113 normal samples were included. In paired analysis, 112 tumor samples and 112 normal samples were included. **D** Pearson correlation analysis between *MYCN* expression and *IGF2BP1* expression in breast cancer patients. **E** MYCN-*IGF2BP1* target was validated by q-ChIP analysis. **F** Mutation strategy of E-Box in *IGF2BP1* promoter for luciferase assay. **G** MYCN-*IGF2BP1* target was validated by luciferase assay analysis. **H** and **I** q-PCR and western blot analysis on IGF2BP1 when MYCN was induced by DOX in MCF-7^MYCN−HA−OV^ cell. **J** Xenograft tumors of 4 groups: scrambled siRNA and Dox treated 231^*MYCN*−HA−OV^ cell; *IGF2BP1* siRNA and Dox treated 231^*MYCN*−HA−OV^ cell; scrambled siRNA 231^*MYCN*−HA−OV^ cell; *IGF2BP1* siRNA 231^*MYCN*−HA−OV^ cell. **K** and **L** Xenograft tumor weight and volume were measured. **M** q-PCR analysis on *MIR210HG* after MYCN was induced by Dox treatment in MCF-7^*MYCN*−HA−OV^ cell. **N** Western blot analysis on IGF2BP1 with indicated treatments. **O** q-PCR analysis on *MIR210HG* and miR-210 after treated with DOX or *IGF2BP1* siRNA as indicated in MCF-7^*MYCN*−HA−OV^ cell. **P** Pearson correlation analysis between MYCN expression and MIR210HG expression in breast cancer patients. **p < 0.01, ***p < 0.001

MYC family has been identified playing critical roles in tumorigenesis by serving as transcription factor, which activates numerous oncogenes by directly binding E-box. In addition, previous studies showed MYC is also regulated by m6A modification. Here, we focused on the transcription ability of MYC family on m6A modification regulators. From Cistrome DB tool, we confirmed that there was one E-box binding motif (CACGTG) under the MYCN enriched peaks in *IGF2BP1* promoter (Fig. 7A), which indicated MYCN is a potential transcription factor of *IGF2BP1*. Clinical relevance analysis revealed that *MYCN* was up-regulated in tumor tissues (Fig. 7B and C). In addition, the expression of *IGF2BP1* was identified positively correlated to *MYCN* expression in breast cancer (Fig. 7D). Therefore, combining previous studies, *MYCN* is an oncogene in breast cancer. Next, we performed q-ChIP analysis to validate the target between MYCN and *IGF2BP1*. Indeed, *IGF2BP1* was enriched in the MYCN occupancy site (Fig. 7E). Then luciferase reporter assay was performed following the indicated mutation strategy in *IGF2BP1* promoter (Fig. 7F). Mutation in binding site resulted in a significantly repressed luciferase activity (Fig. 7G) in generated MYCN overexpression MCF-7 cell with inducible vector (MCF-7^*MYCN*−HA−OV^). q-PCR analysis showed DOX induced *MYCN* increased *IGF2BP1* expression (Fig. 7H) in a time dependent manner. The similar results can also be observed in western blot analysis (Fig. 7I). Of note, ectopic *MYCN* expression resulted in increased tumor volume and weight in xenograft model but *IGF2BP1* silencing largely abrogated this effect (Fig. 7J–L). In summary, MYCN functions as a transcription factor of *IGF2BP1*, which induces I*GF2BP1* expression and promotes breast cancer progression.

Since we confirmed that *IGF2BP1* is a direct target of MYCN, next, the potential regulation between MYCN and *MIR210HG* was investigated. q-PCR analysis showed that MYCN activation increased *MIR210HG* expression (Fig. 7M). To investigate whether IGF2BP1 mediated the function of MYCN on *MIR210HG*, *IGF2BP1* was knocked down achieved by siRNA pool in MCF-7^*MYCN*−HA−OV^ cell (Fig. 7N). MYCN activation induced *MIR210HG* expression but this effect was largely abrogated by *IGF2BP1* knocking down (Fig. 7O). The similar results can also be observed in miR-210 expression pattern (Fig. 7O). In addition, clinical relevance data showed that *MIR210HG* expression was positively correlated to *MYCN* expression in breast cancer (Fig. 7P). Therefore, both miR-210 and *MIR210HG* are regulated by MYCN, which is mediated by IGF2BP1.

## Discussion

**Fig. 8:**
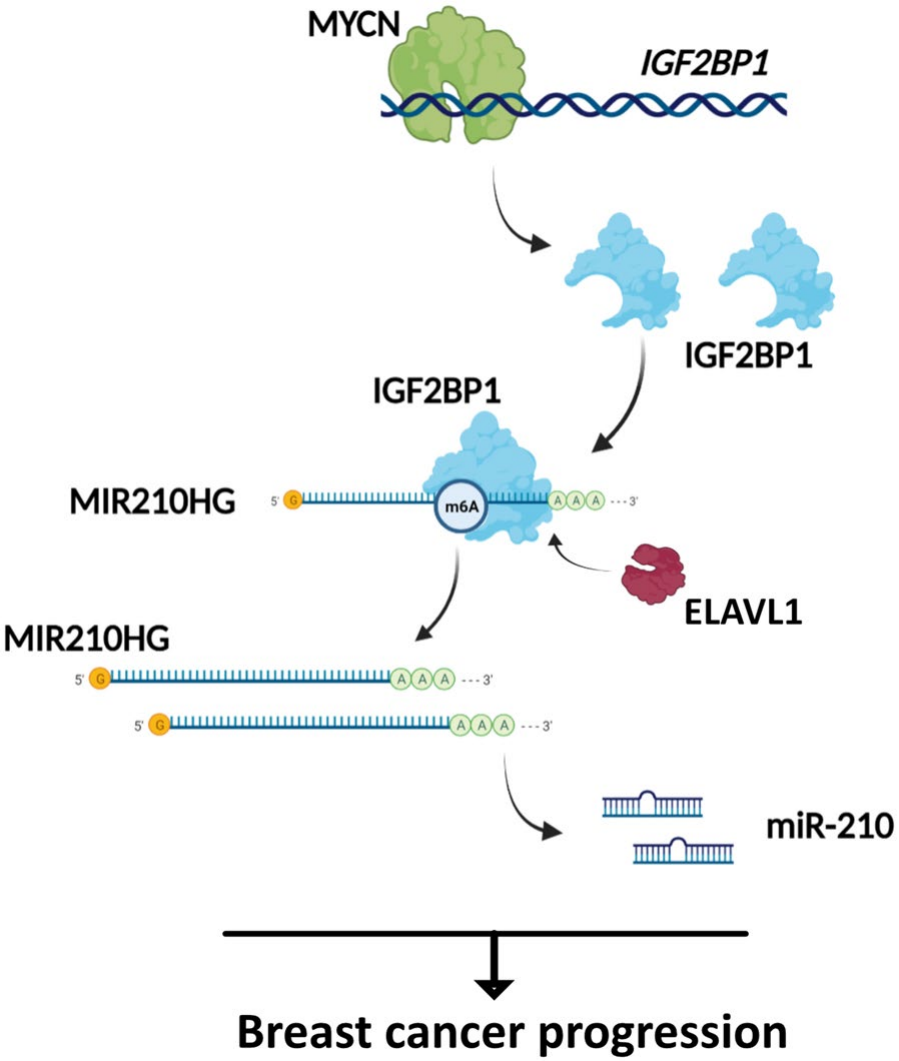
Regulation model of MYCN/IGF2BP1/*MIR210HG* axis in breast cancer progression. *MIR210HG* is an oncogenic lncRNA which promotes breast cancer progression. miR-210 mediates *MIR210HG* function and IGF2BP1 mediated m6A regulation induces *MIR210HG* expression. Furthermore, *IGF2BP1* is a direct target of MYCN. *MIR210HG*/miR-210 is also induced by MYCN dependent on IGF2BP1

In this study, we demonstrated that *MIR210HG* was induced by IGF2BP1 through m6A modification in breast cancer. Downstream miR-210 was identified critical for *MIR210HG* function. In addition, MYCN was characterized as a transcription factor that activated *IGF2BP1* expression and regulated *MIR210HG* (summarized in Fig. 8). Emerging evidence suggests that m6A modification is involved in proliferation, metastasis and tumorigenesis of breast cancer [17]. IGF2BP1 is an RNA binding protein functions as a “reader” in m6A modification. Previous studies have shown IGF2BP1 contributes to the stabilization of IGF2BP-RNA complexes via KH1/2 domain [18]. Interestingly, *MYC* mRNA is stabilized by IGF2BP1 [16], which results in an oncogenic role of IGF2BP1. Here, we confirmed that the lncRNA *MIR210HG* is increased by IGF2BP1 through m6A methylation and direct binding. Actually, *MIR210HG* has been reported to function as an oncogene in different cancers [19–21]. Through sponging miRNAs, *MIR210HG* promotes tumor proliferation, metastasis and in some cases, is involved in energy metabolism in cancer. In line with the results of previous researches, here we validated *MIR210HG* promoted proliferation and metastasis of breast cancer by using both cellular experiments and xenograft model. More importantly, its encoded miR-210 mediated *MIR210HG* function, which suggests that *MIR210HG*/miR-210 axis is critical in breast cancer progression. However it is still unclear the changes of gene expression when *MIR210HG* is completely knocked out in vivo. Therefore, based on our current results, it is interesting to include transgenic mice to perform RNA-Seq analysis with different *MIR210HG* status. In breast cancer, other studies reveal miR-210 is regulated by hypoxia via HIF-1α/VHL transcriptional system [22]. Therefore, we suppose that *MIR210HG*/miR-210 is also involved hypoxia regulation but it needs further validation.

In this study, it is the first time that *MIR210HG*-m6A modification to be investigated. The confirmed interaction of IGF2BP1 and *MIR210HG* provides an extended explanation of the molecular mechanism in breast cancer progression. Recent studies identify that m6A modification is one of the important ways on oncogene regulation. As a m6A reader, IGF2BP1 stabilizes mRNA such as *SRF* to promote cell growth and invasion in ovarian and liver cancer [23]. In our study, *MIR210HG* was stabilized by IGF2BP1, indicating *MIR210HG* is induced by two ways via m6A modification. Previous studies have identified that several co-factors of IGF2BPs enhance the stability of mRNA [16]. Here, we confirmed that ELAVL1, one of the co-factors of IGF2BPs, is involved in maintaining *MIR210HG* together with IGF2BP1. Therefore, *MIR210HG* stability is controlled by a protein complex containing both IGF2BP1 and ELAVL1. Nevertheless, further studies are needed to clarify the reason that ELAVL1 is induced in breast cancer. In addition, there were other m6A mediators including YTHDFs predicted to be involved in *MIR210HG* m6A regulation. Therefore, *MIR210HG* is possibly located in a more complicated m6A regulation mediated by multiple regulators. However, it needs further validation to describe the whole landscape of *MIR210HG* m6A modification.

Interestingly, we further confirmed that MYCN is a transcription factor of *IGF2BP1*, which induces IGF2BP1 and downstream *MIR210HG*/miR-210 expression. Many studies have shown MYCN and c-MYC directly regulate genes as transcription factors regulating multiple biological or pathological processes such as cell growth, cell cycle, metastasis and DNA damage [24]. Hence, IGF2BP1-*MIR210HG*/miR-210 axis provides an alternative explanation that MYCN is an oncogene in breast cancer. Of note, MYC has been identified to be regulated and stabilized by IGF2BP1 [16]. Therefore, the stimulation of MYC-IGF2BP1 may form a positive feedback loop, which may further induce *MIR210HG*/miR-210 downstream. The positive hypothesis is still needed further experimental validation. Collectively, MYCN/IGF2BP1 target provides a prerequisite condition for *MIR210HG* m6A modification by IGF2BP1. Meanwhile, IGF2BP1/*MIR210HG*/miR-210 regulatory axis mediates the tumorigenesis role of MYCN in breast cancer.

In summary, *MIR210HG* functions as an oncogenic lncRNA in breast cancer, which is also mediated by its encoded miR-210. In addition, both IGF2BP1 and ELAVL1 enhance the stability of *MIR210HG*, which contributes to the progression of breast cancer. Interestingly, *IGF2BP1* is directly activated by MYCN, which explains the oncogenic role of MYCN. These findings clarify the m6A regulation related molecular mechanism of breast cancer progression. The MYCN/IGF2BP1/*MIR210HG* axis may serve as an alternative molecular mechanism of breast cancer progression.

## Supplementary Information

**Additional file 1: File 1. Table S1.** Primers used in q-PCR analysis. **Table S2.** Primary antibodies used in western blot, q-ChIP and IHC. **Table S3.** Primers used in liciferase assay. **Table S4.** Primers used in qChIP analysis. **File 2.** Original gels.

## Data Availability

All data generated or analyzed during this study are included in this published article and its supplementary information files.

## Abbreviations

TCGA: The Cancer Genome Atlas
MIR210HG: MIR210 host gene
IGF2BP1: Insulin like growth factor 2 mRNA binding protein 1
MYCN: MYCN proto-oncogene, bHLH transcription factor
BRCA: Breast cancer
q-ChIP: Chromatin immunoprecipitation-quantitative PCR

## References

[1] Ghoncheh M, Pournamdar Z, Salehiniya H (2016). Incidence and mortality and epidemiology of breast cancer in the world. Asian Pac J Cancer Prev.

[2] DeSantis CE, Ma J, Gaudet MM, Newman LA, Miller KD, Goding Sauer A (2019). Breast cancer statistics, 2019. CA Cancer J Clin.

[3] Thakur KK, Kumar A, Banik K, Verma E, Khatoon E, Harsha C (2021). Long noncoding RNAs in triple-negative breast cancer: a new frontier in the regulation of tumorigenesis. J Cell Physiol.

[4] Heidari R, Akbariqomi M, Asgari Y, Ebrahimi D, Alinejad-Rokny H (2021). A systematic review of long non-coding RNAs with a potential role in breast cancer. Mutat Res.

[5] Aliperti V, Skonieczna J, Cerase A (2021). Long non-coding RNA (lncRNA) roles in cell biology, neurodevelopment and neurological disorders. Noncoding RNA..

[6] Min W, Dai D, Wang J, Zhang D, Zhang Y, Han G (2016). Long noncoding RNA miR210HG as a potential biomarker for the diagnosis of glioma. PLoS ONE.

[7] Ma J, Kong FF, Yang D, Yang H, Wang C, Cong R (2021). lncRNA MIR210HG promotes the progression of endometrial cancer by sponging miR-337-3p/137 via the HMGA2-TGF-beta/Wnt pathway. Mol Therapy Nucleic Acids.

[8] Du Y, Wei N, Ma R, Jiang SH, Song D (2020). Long noncoding RNA MIR210HG promotes the Warburg effect and tumor growth by enhancing HIF-1alpha translation in triple-negative breast cancer. Front Oncol.

[9] Li XY, Zhou LY, Luo H, Zhu Q, Zuo L, Liu GY (2019). The long noncoding RNA MIR210HG promotes tumor metastasis by acting as a ceRNA of miR-1226-3p to regulate mucin-1c expression in invasive breast cancer. Aging (Albany NY).

[10] Dominissini D, Moshitch-Moshkovitz S, Schwartz S, Salmon-Divon M, Ungar L, Osenberg S (2012). Topology of the human and mouse m6A RNA methylomes revealed by m6A-seq. Nature.

[11] Ma S, Chen C, Ji X, Liu J, Zhou Q, Wang G (2019). The interplay between m6A RNA methylation and noncoding RNA in cancer. J Hematol Oncol.

[12] Visvanathan A, Patil V, Arora A, Hegde AS, Arivazhagan A, Santosh V (2018). Essential role of METTL3-mediated m(6)A modification in glioma stem-like cells maintenance and radioresistance. Oncogene.

[13] Wang P, Doxtader KA, Nam Y (2016). Structural basis for cooperative function of Mettl3 and Mettl14 methyltransferases. Mol Cell.

[14] Niu Y, Lin Z, Wan A, Chen H, Liang H, Sun L (2019). RNA N6-methyladenosine demethylase FTO promotes breast tumor progression through inhibiting BNIP3. Mol Cancer.

[15] Dang Q, Shao B, Zhou Q, Chen C, Guo Y, Wang G (2021). RNA N (6)-methyladenosine in cancer metastasis: roles, mechanisms, and applications. Front Oncol.

[16] Huang H, Weng H, Sun W, Qin X, Shi H, Wu H (2018). Recognition of RNA N(6)-methyladenosine by IGF2BP proteins enhances mRNA stability and translation. Nat Cell Biol.

[17] Lan Q, Liu PY, Haase J, Bell JL, Huttelmaier S, Liu T (2019). The critical role of RNA m(6)A methylation in cancer. Cancer Res.

[18] Huang X, Zhang H, Guo X, Zhu Z, Cai H, Kong X (2018). Insulin-like growth factor 2 mRNA-binding protein 1 (IGF2BP1) in cancer. J Hematol Oncol.

[19] Yu T, Li G, Wang C, Gong G, Wang L, Li C (2021). MIR210HG regulates glycolysis, cell proliferation, and metastasis of pancreatic cancer cells through miR-125b-5p/HK2/PKM2 axis. RNA Biol.

[20] Bu L, Zhang L, Tian M, Zheng Z, Tang H, Yang Q (2020). LncRNA MIR210HG facilitates non-small cell lung cancer progression through directly regulation of miR-874/STAT3 Axis. Dose Response.

[21] Wang AH, Jin CH, Cui GY, Li HY, Wang Y, Yu JJ (2020). MIR210HG promotes cell proliferation and invasion by regulating miR-503-5p/TRAF4 axis in cervical cancer. Aging (Albany NY).

[22] Camps C, Buffa FM, Colella S, Moore J, Sotiriou C, Sheldon H (2008). hsa-miR-210 is induced by hypoxia and is an independent prognostic factor in breast cancer. Clin Cancer Res.

[23] Muller S, Glass M, Singh AK, Haase J, Bley N, Fuchs T (2019). IGF2BP1 promotes SRF-dependent transcription in cancer in a m6A- and miRNA-dependent manner. Nucleic Acids Res.

[24] Liu Z, Chen SS, Clarke S, Veschi V, Thiele CJ (2020). Targeting MYCN in pediatric and adult cancers. Front Oncol.

