# Supplementary material for: MIR210HG promotes breast cancer progression by IGF2BP1 mediated m6A modification
Source: Cell Biosci. 2022 Mar 28;12:38. doi: 10.1186/s13578-022-00772-z (PMC8962467; doi:10.1186/s13578-022-00772-z)

**Additional files**

**File 1: Additional tables.**

**Table S1**. Primers used in q-PCR analysis.

**Table S2.** Primary antibodies used in western blot, q-ChIP and IHC.

**Table S3.** Primers used in luciferase assay.

**Table S4.** Primers used in qChIP analysis.

**File 2: Original gels.**

**File 1**

**Table S1**. Primers used in q-PCR analysis.

| Gene | Sequences (5’-3’) |
| --- | --- |
| MIR210HG Forward | AGGCAGATTTAGTGGACGCC |
| MIR210HG Reverse | ACAGCCTTTCTCAGGTGCAG |
| IGF2BP1 Forward | GCGGCCAGTTCTTGGTCAA |
| IGF2BP1 Reverse | TTGGGCACCGAATGTTCAATC |
| E-cadherin Forward | CGAGAGCTACACGTTCACGG |
| E-cadherin Reverse | GGGTGTCGAGGGAAAAATAGG |
| Vimentin Forward | GACGCCATCAACACCGAGTT |
| Vimentin Reverse | CTTTGTCGTTGGTTAGCTGGT |
| ELAVL1 Forward | AACTACGTGACCGCGAAGG |
| ELAVL1 Reverse | CGCCCAAACCGAGAGAACA |
| MATR3 Forward | ATCAATGGAGCAAGTCACAGTC |
| MATR3 Reverse | TGCAACATGAATGGATCACCC |
| PABPC1 Forward | CAGGCTCACCTCACTAACCAG |
| PABPC1 Reverse | GGTAGGGGTTGATTACAGGGT |

**Table S2.** Primary antibodies used in western blot, q-ChIP, co-IP and IHC.

| Epitope | Catalog No. | Company | Use | Dilution |
| --- | --- | --- | --- | --- |
| E-cadherin | ab40772 | Abcam | WB | 1:1000 |
| Vimentin | ab20346 | Abcam | WB | 1:1000 |
| HA-tag | 51064-2-AP | Proteintech | WB | 1:2500 |
| IGF2BP1 | ab184305 | Abcam | WB, IHC, co-IP | 1:1000, 1:4000, 1:500 |
| MYCN | ab227822 | Abcam | q-ChIP | 5 μg |
| Ki-67 | ab15580 | Abcam | IHC | 0.2 μg/ml |
| β-actin | MA5-11866 | Thermo Fisher | WB | 1:2000 |
| ELAVL1 | ab200342 | Abcam | WB | 1:1000 |
| IgG | Ab172730 | Abcam | IP | 1:500 |

**Table S3.** Primers used in luciferase assay.

| Genes | Sequences (5’-3’) |
| --- | --- |
| IGF2BP1 WT Forward | GGCGCCATCCCAGCCTCC |
| IGF2BP1 WT Reverse | CAGAGGTCGCGGGAGAGTTC |
| IGF2BP1 MUT Forward | TGTCACggcaatGCTTCTCCTTTTTTTTTTTTTTTTTT |
| IGF2BP1 MUT Reverse | AGAAGCattgccGTGACAGCCCCCACCCAC |

**Table S4.** Primers used in qChIP analysis.

| Gene | Sequences (5’-3’) |
| --- | --- |
| IGF2BP1 Forward | TGCCGCACCGCCCCAGTT |
| IGF2BP1 Reverse | GGCTGGTGTGTCACGTTTCTC |

**File 2: Original gels.**


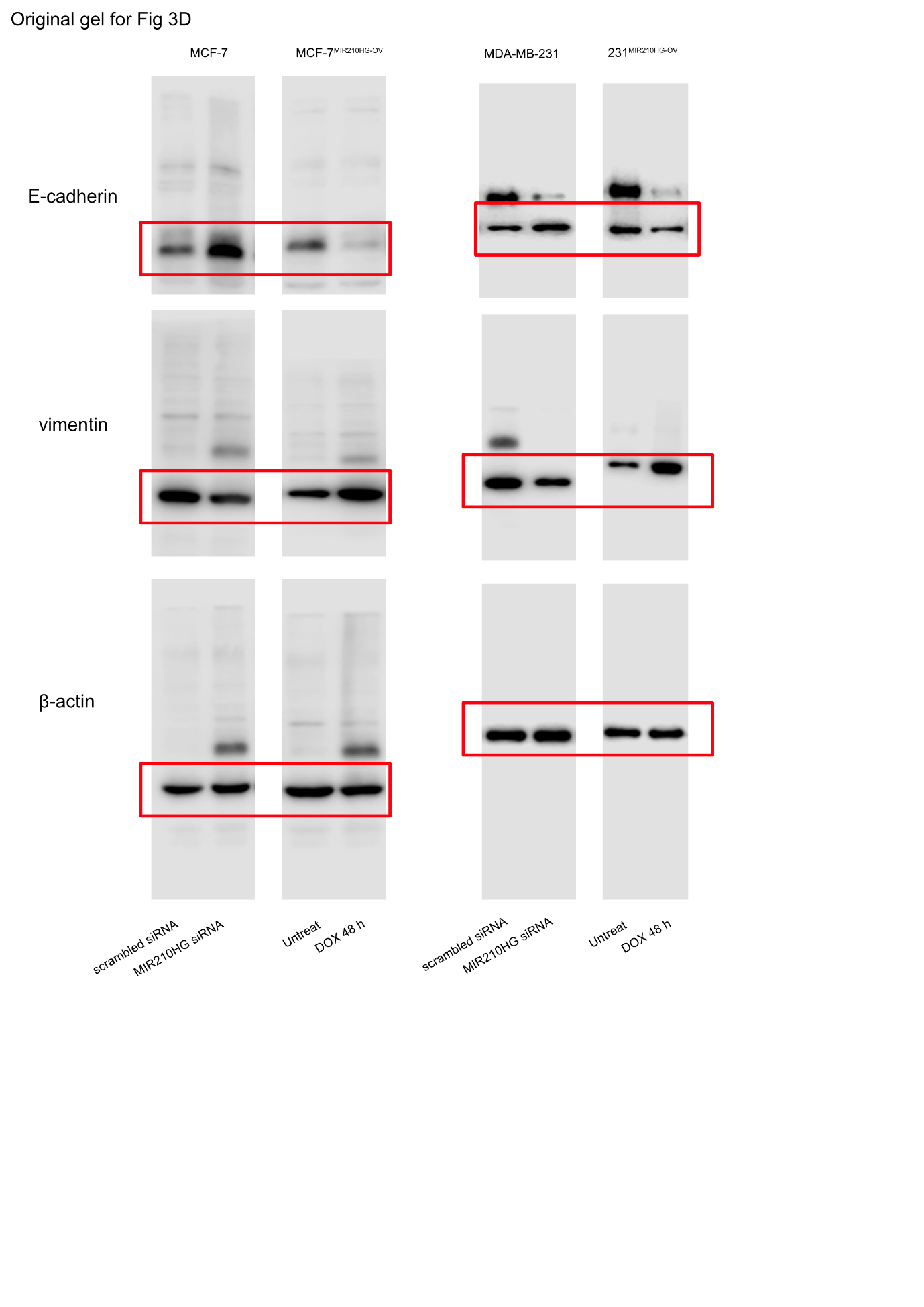


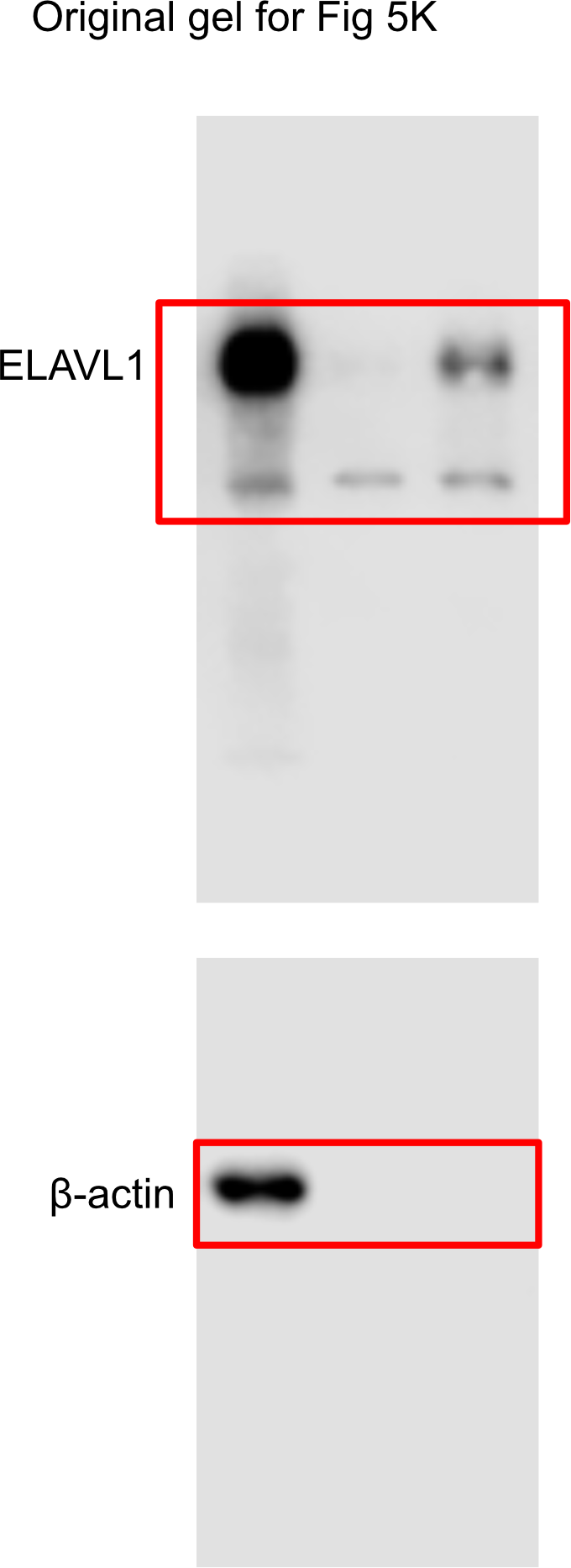


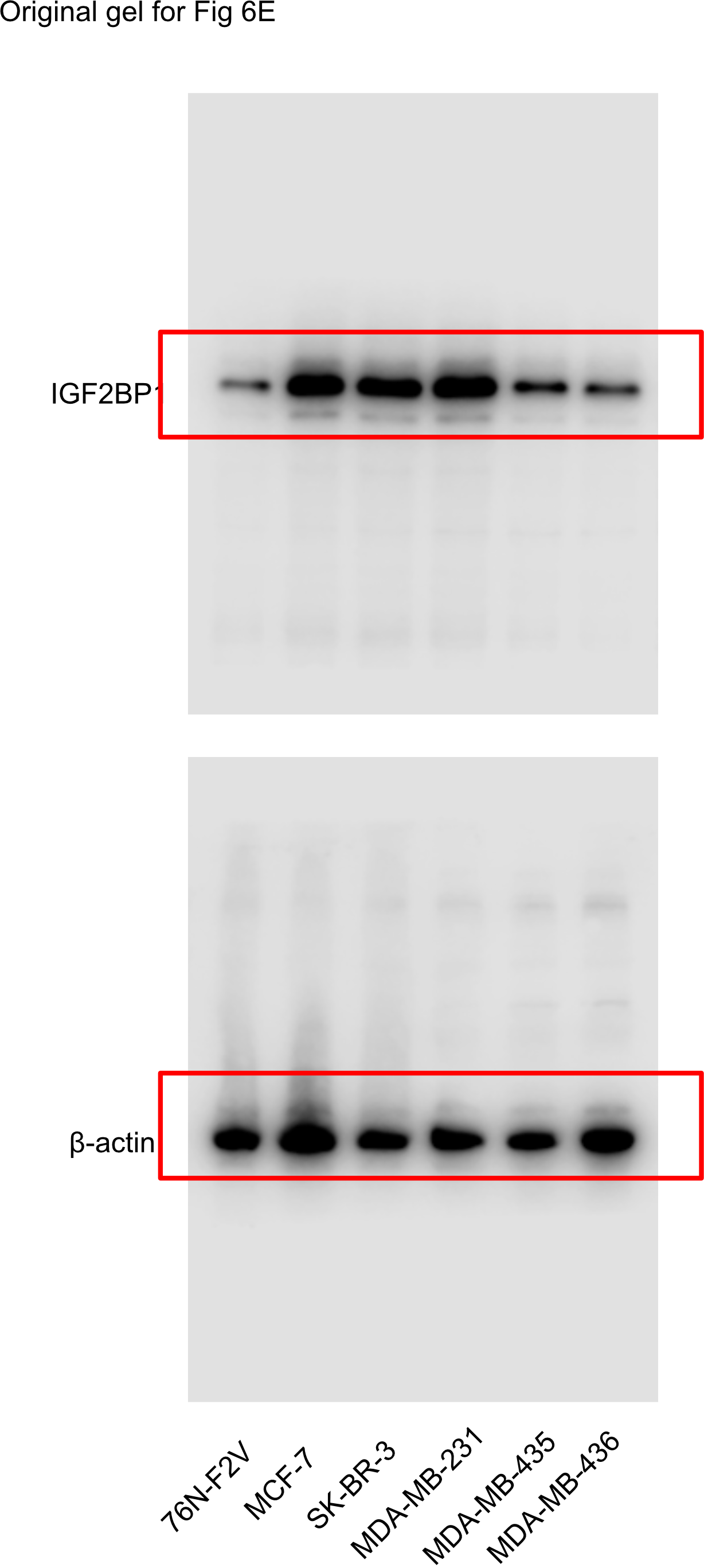


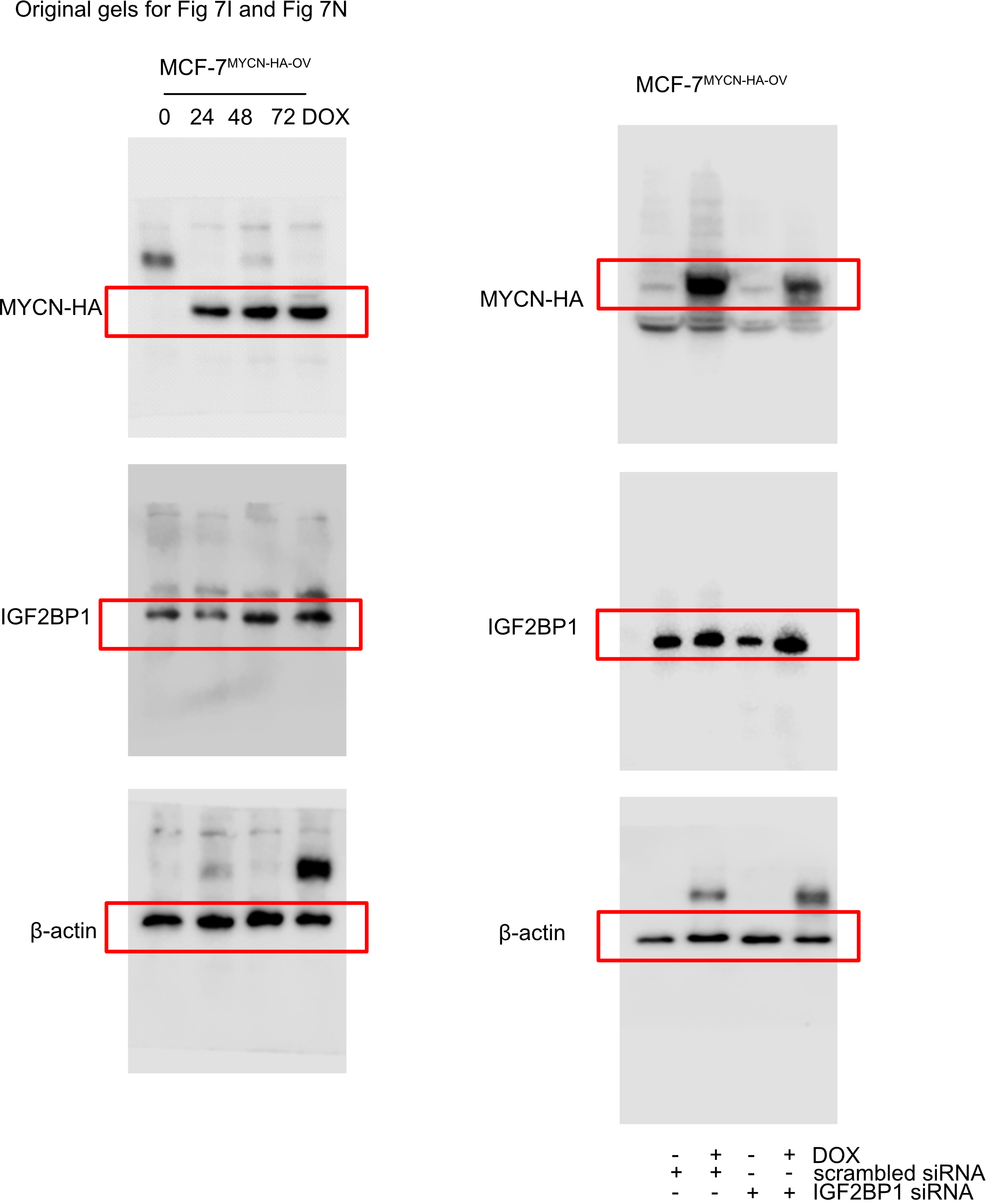

Supplement: Supplementary file 1 — Additional file 1: File 1. Table S1. Primers used in q-PCR analysis. Table S2. Primary antibodies used in western blot, q-ChIP and IHC. Table S3. Primers used in liciferase assay. Table S4. Primers used in qChIP analysis. File 2. Original gels. [file 13578_2022_772_MOESM1_ESM.docx]
